# Chemometric Analysis for the Prediction of Biochemical Compounds in Leaves Using UV-VIS-NIR-SWIR Hyperspectroscopy

**DOI:** 10.3390/plants12193424

**Published:** 2023-09-28

**Authors:** Renan Falcioni, João Vitor Ferreira Gonçalves, Karym Mayara de Oliveira, Caio Almeida de Oliveira, Amanda Silveira Reis, Luis Guilherme Teixeira Crusiol, Renato Herrig Furlanetto, Werner Camargos Antunes, Everson Cezar, Roney Berti de Oliveira, Marcelo Luiz Chicati, José Alexandre M. Demattê, Marcos Rafael Nanni

**Affiliations:** 1 Graduate Program in Agronomy, Department of Agronomy, State University of Maringá, Av. Colombo 5790, Maringá 87020-900, PR, Brazil; pg403805@uem.br (J.V.F.G.); eng.karymoliveira@gmail.com (K.M.d.O.); pg55482@uem.br (C.A.d.O.); reisamanda89@gmail.com (A.S.R.); wcantunes@uem.br (W.C.A.); rboliveira@uem.br (R.B.d.O.); mlchicati@uem.br (M.L.C.); mrnanni@uem.br (M.R.N.); 2 Embrapa Soja (National Soybean Research Centre–Brazilian Agricultural Research Corporation), Londrina 86001-970, PR, Brazil; luis.crusiol@colaborador.embrapa.br; 3 Institute of Food and Agricultural Sciences, University of Florida, Gainesville, FL 32611, USA; re.herrigfurlane@ufl.edu; 4 Department of Agricultural and Earth Sciences, University of Minas Gerais State, Passos 37902-108, MG, Brazil; everson.cezar@uemg.br; 5 Department of Soil Science, Luiz de Queiroz College of Agriculture, University of São Paulo, Av. Pádua Dias 11, Piracicaba 13418-260, SP, Brazil; jamdemat@usp.br

**Keywords:** algorithms, modelling, partial least square regression, plant phenotyping, reflectance data, vegetation indices, wavelengths

## Abstract

Reflectance hyperspectroscopy is recognised for its potential to elucidate biochemical changes, thereby enhancing the understanding of plant biochemistry. This study used the UV-VIS-NIR-SWIR spectral range to identify the different biochemical constituents in Hibiscus and Geranium plants. Hyperspectral vegetation indices (HVIs), principal component analysis (PCA), and correlation matrices provided in-depth insights into spectral differences. Through the application of advanced algorithms—such as PLS, VIP, *i*PLS-VIP, GA, RF, and CARS—the most responsive wavelengths were discerned. PLSR models consistently achieved R^2^ values above 0.75, presenting noteworthy predictions of 0.86 for DPPH and 0.89 for lignin. The red-edge and SWIR bands displayed strong associations with pivotal plant pigments and structural molecules, thus expanding the perspectives on leaf spectral dynamics. These findings highlight the efficacy of spectroscopy coupled with multivariate analysis in evaluating the management of biochemical compounds. A technique was introduced to measure the photosynthetic pigments and structural compounds via hyperspectroscopy across UV-VIS-NIR-SWIR, underpinned by rapid multivariate PLSR. Collectively, our results underscore the burgeoning potential of hyperspectroscopy in precision agriculture. This indicates a promising paradigm shift in plant phenotyping and biochemical evaluation.

## 1. Introduction

Over the past few years, hyperspectral spectroscopy has become prominent, revolutionising botanical and agronomic research and bridging the intricacies of plant biology with state-of-the-art technological advancements [1,2]. Revolutionary advances in remote sensing technology, particularly in hyperspectral non-imaging and imaging, have expanded the frontiers of precision agriculture, environmental monitoring, and plant physiology research. Spanning the regions from ultraviolet to shortwave infrared (UV-VIS-NIR-SWIR), this technique boasts an unparalleled depth, offering comprehensive spectral analysis. This enables researchers to delve deeper into plants’ biochemical and physiological processes, revealing detailed snapshots of their health, structure, and function at various scales [3,4]. Hyperspectral technology provides an unparalleled opportunity to decipher plant health, physiological state, and response to environmental stresses [5].

*Hibiscus rosa-sinensis* L. (Hibiscus) and *Pelargonium zonale* L’Hér. Ex. Aiton (Geranium) stands out in the floral world for its vibrant hues and ornamental appeal and its multifaceted utility in traditional medicine and potential pharmaceutical applications [6]. The intricate biochemistry of these plants and their ecological significance necessitate detailed and non-invasive investigative methodologies to foster our understanding and enable informed interventions in their cultivation and application [7].

Chemometric analysis using UV-VIS-NIR-SWIR hyperspectral data is an emerging approach to unravel the underlying relationship between spectral reflectance patterns and biochemical constituents of plant leaves [8,9]. By integrating spectral data with advanced statistical methods, such as principal component analysis (PCA) and selecting wavelengths and bands most responsively, it becomes feasible to detect subtle differences in leaf biochemistry, even before they manifest as visible symptoms [10].

Despite the evident capabilities of hyperspectral spectroscopy, these challenges persist. One of the principal roadblocks is the sheer volume and complexity of the obtained data. This wealth of information, although invaluable, requires advanced computational algorithms for accurate interpretation and application [11]. The intertwining of artificial intelligence, machine learning, and hyperspectral data holds promise for navigating this vast data terrain, offering nuanced insights previously out of reach [1,12].

Conventional methodologies that are fundamental to our understanding often have inherent limitations. Invasive techniques that frequently require destructive sampling compromise the sample’s integrity and distort the resulting observations. This challenges the reliability and reproducibility of the conclusions drawn from such methods [3,13]. The emergence of non-invasive hyperspectral techniques offers a promising solution to these challenges. They redefined the approach to research in this domain, minimising both the ecological and economic impacts of investigations [14,15]. Furthermore, these advanced techniques can accurately assess plant components such as pigments, antioxidant capacity, and structural molecules. This comprehensive insight allows for the development of strategies for the utilisation of these plants.

The interplay between plant molecule-based reflectance hyperspectroscopy and the ability of hyperspectral spectroscopy to provide a window into this intricate relationship, particularly in the context of climate change and evolving environmental stressors, makes it an indispensable tool in modern botanical research toolkits [16,17,18].

Our study aims to integrate advanced computational intelligence, classification algorithms, and selected wavelengths and bands to extract valuable information from UV-VIS-NIR-SWIR hyperspectral data on plant growth in greenhouses. We hypothesised that this approach would enable the non-destructive assessment, classification, calibration, and prediction of biochemical parameters, such as chlorophylls, carotenoids, flavonoids, radical scavenging, lignin, and cellulose molecules, with high accuracy and precision. This study aimed to demonstrate that reflectance hyperspectral remote sensing technology can provide alternative and rapid methods for estimating biochemical attributes in leaves.

## 2. Results

### 2.1. Descriptive Biochemical Parameters

The biochemical parameters of *Hibiscus rosa-sinensis* L. (Hibiscus) and *Pelargonium zonale* (L.) L’Hér. Ex. Aiton (Geranium) leaves were comprehensively evaluated, with the specifics detailed in Table 1. From the 200 samples, area-based measurements exhibited an average Chl*a* concentration of 1322.8 mg m^−2^ (CV: 29.0%), while the mass-based metric revealed an average of 64.2 mg g^−1^ (CV: 27.0%). Chl*b*, at both the area (1012.0 mg m^−2^) and mass scales (46.0 mg g^−1^), recorded CVs of 47.0% and 31.4%, respectively. Flavonoids (Flv) indicated pronounced variability, with the area-based CV at 31.8% and the mass-based figure reaching 60.0%. Notably, the DPPH parameter remained the most consistent, with a CV of 2.9%. The structural constituents of lignin and cellulose exhibited CVs of 19.7% and 37.4%, respectively. These findings offer comprehensive insight into the biochemical attributes of the evaluated plant species, considering both area- and mass-based metrics for leaves.

### 2.2. Pearson’s Correlation and Principal Component Analysis of Hibiscus and Geranium Leaves

**Figure 1:**
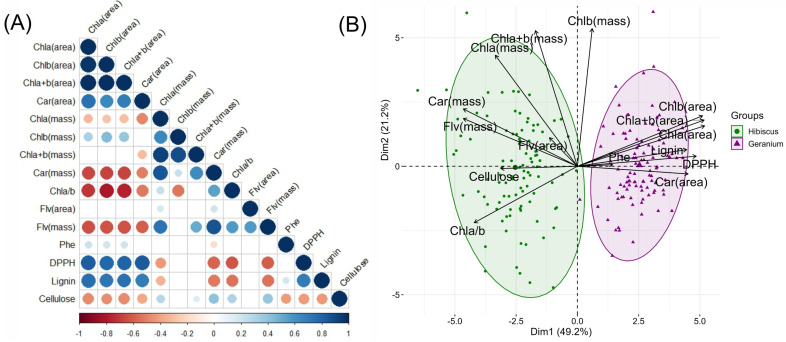
Pearson’s correlation and principal component analysis of the plant samples. (**A**) Biochemical molecule measurements using Pearson’s correlation, ranging from −1 to +1. (**B**) Principal Component Analysis (PCA) of samples from both the Hibiscus and Geranium groups plotted along Dimension 1 (Dim 1) and Dimension 2 (Dim 2). The measured extracts and growth variables included chlorophyll *a*-based area (Chl*a*(area)), chlorophyll *b*-based area (Chl*b*(area)), combined chlorophyll *a*+*b*-based area (Chl*a*+*b*(area)), carotenoid-based area (Car(area)), chlorophyll *a*-based mass (Chl*a*(mass)), chlorophyll *b*-based mass (Chl*b*(mass)), combined chlorophyll *a*+*b*-based mass (Chl*a*+*b*(mass)), carotenoid-based mass (Car(mass)), chlorophyll *a*/*b* ratio (Chl*a*/*b*), flavonoid-based area (Flv(area)), flavonoid-based mass (Flv(mass)), phenolic compounds (Phe), radical scavenging activity (DPPH), lignin, and cellulose. (n = 200).

A correlation matrix and principal component analysis were used to analyse the complex interplay of biochemical parameters in *Hibiscus rosa-sinensis* L. and *Pelargonium zonale* (L.) L’Hér. Ex. Aiton, as shown in Figure 1. Lignin concentrations exhibited strong associations with Chl*a* (mg m^−2^) r = 0.758, Chl*a*+*b* (mg m^−2^) r = 0.742, and Chl*b* (mg m^−2^) r = 0.723. The radical scavenging potential, depicted by DPPH, showed positive correlations with carotenoids based on area measurements (r = 0.831) and Chl*a* (mg m^−2^) (r = 0.822), while negative correlations were observed with Flv (μmol g^−1^) (r = −0.605) and the Chl*a*/*b* ratio (r = −0.620). In this sense, carotenoids, in terms of both area- and mass-based measurements, demonstrated significant correlations with other parameters. The area-based carotenoid measurements were positively correlated with DPPH (r = 0.831) and Chl*a* (mg m^−2^) (r = 0.746). In contrast, mass-based measurements displayed strong positive relationships with Flv (μmol g^−1^) (r = 0.864) and Chl*a* (mg g^−1^) (r = 0.859) (*p* < 0.01) (Figure 1A).

Principal component analysis (PCA) was performed to further delve into the spectral data, and the results are shown in Figure 1B. The two primary dimensions, Dimension 1 (Dim 1) and Dimension 2 (Dim 2), collectively accounted for a significant 63.4% variance—42.2% attributed to Dim 1 and 21.2% to Dim 2. The evident clustering in the PCA plot highlights the inherent spectral differences and unique biochemical compositions of Hibiscus and Geranium. In addition, the vectors demonstrate a higher correlation for mass-based biochemical compounds (Car, Chl*a*, Chl*a*+*b*, Flv) for Hibiscus and area-based biochemical compounds (Chl*b*, Chl*a*+*b* Car, Chl*a*) for Geranium plants. This in-depth analysis aimed to identify specific compounds and their interactions, providing insights into their distinctive biochemical and structural attributes (Figure 1).

### 2.3. Spectral Reflectance and Principal Component Analysis of Hibiscus and Geranium Leaves

**Figure 2:**
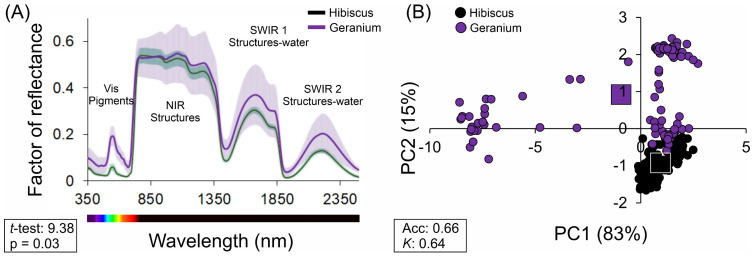
Spectral reflectance and PCA of *Hibiscus rosa-sinensis* L. (Hibiscus) and *Pelargonium zonale* (L.) L’Hér. Ex. Aiton (Geranium) leaves range in size from 350 to 2500 nm. The dotted lines at 700 nm and 1300 nm demarcate the transitions from VIS (visible) to NIR (near-infrared) and from NIR to shortwave infrared (SWIR) bands, respectively. (**A**) Spectral reflectance graph with Hibiscus (green) and Geranium (purple) represented. (**B**) PCA of the two species. Values for Accuracy (Acc) and Kappa (*K*) coefficients are reported in boxes. Each data point represents the mean of measurements. The standard deviation was omitted for clarity. Statistical significance between Hibiscus and Geranium was assessed using a *t*-test (9.38), with *p*-values of 0.03 reported. (n = 200).

For distinct maximum factors, based on the vectors analysed, the hyperspectral reflectance values for *Hibiscus rosa-sinensis* L. and *Pelargonium zonale* (L.) L’Hér. Ex. Aiton was evaluated across the UV-VIS-NIR-SWIR bands with a spectral resolution of 1 nm (Figure 2). Within this spectrum, clear demarcations at 700 nm and 1300 nm indicate transitions from the visible (VIS) spectrum to the near-infrared (NIR) and from NIR to the shortwave infrared (SWIR) bands, respectively. In addition, a *t* test comparison yielded a value of 9.38 with a corresponding *p* value of 0.03, signifying marked differences in the biochemical attributes of the leaves between the two species (Figure 2A).

To further elucidate these differences, principal component analysis (PCA) was employed for the hyperspectral curves (Figure 2B). The first principal component (PC1) accounted for 83% of the total variance, while the second (PC2) represented 15%. The mean PCA value for Hibiscus on PC1 was 0.932, in contrast to the Geranium plant’s mean of ™0.923, with an accuracy of 0.66 and Kappa coefficient of 0.64, emphasising the distinct spectral characteristics of each species. This analysis was conducted to identify the specific compounds in the leaves of both plants.

The observed spectral differences between the two species highlight their unique biochemical and structural leaf optical properties.

**Figure 3:**
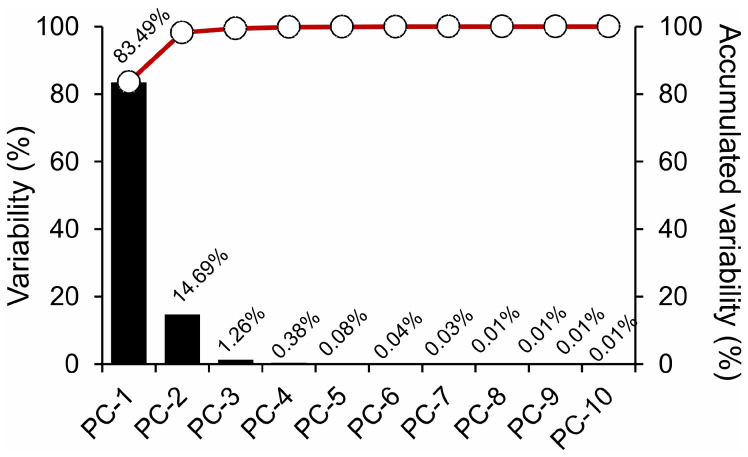
Principal Component Analysis (PCA) illustrating variability in hyperspectral data for *Hibiscus rosa-sinensis* L. (Hibiscus) and *Pelargonium zonale* (L.) L’Hér. Ex. Aiton (Geranium) plants. Black bars represent individual PC variability, whereas red circles indicate cumulative variability.

Based on Figure 3, the subsequent components, namely, PC-3, PC-4, and PC-5, contributed minimally, accounting for just over 1% of the total variance, and PC-6 and PC-10 contributed 0.05% of the data. The cumulative variability across the components was visually depicted with red circles, confirming the dominance of PC-1 and PC-2 in capturing the spectral differences between Hibiscus and Geranium (Figure 2 and Figure 3). This analysis underscored the inherent spectral variability and highlighted the significant contribution of the initial components to variance in the data (Figure 3).

### 2.4. Calibration, Cross-Validation, and Prediction of Biochemical Parameters in Hibiscus and Geranium Leaves

Calibration and cross-validation were undertaken using partial least squares regression (PLSR) to establish the relationships between hyperspectral reflectance data and the biochemical parameters in leaves of *Hibiscus rosa-sinensis* L. and *Pelargonium zonale* (L.) L’Hér. Ex. Aiton (Table 2). During the calibration process, chlorophyll *a*, based on area (Chl*a*+*b*(area)) and mass (Chl*a*+*b*(mass)), presented values (R^2^) of 0.73 and 0.14, respectively. However, upon cross-validation, these values experienced a slight dip, measuring 0.71 and 0.08, respectively. Specifically, for chlorophyll *a* (Chl*a* (mg m^−2^)), a value of 0.73 was observed during calibration, which was adjusted to 0.71 in the cross-validation phase. Chlorophyll *b* (Chl*b* (mg m^−2^)) recorded a value of 0.63 in calibration and 0.60 in cross-validation. Carotenoids considered in terms of area and mass showcased values of 0.86 and 0.49 during calibration, adjusting to 0.85 and 0.43 during cross-validation.

Furthermore, this study also examined other significant biochemical parameters. For instance, the lignin concentration displayed a calibration of 0.74, which was adjusted to 0.71 during the cross-validation phase. However, while the study examined parameters such as the radical scavenging potential of DPPH, cellulose, and Flv, they showed significantly higher values. Nevertheless, the comprehensive results of these metrics require further research or data acquisition to establish the contribution of the most associated bands (Table 2).

Such patterns, spanning chlorophyll to lignin, underscore the robustness of the PLSR model base area and are minor for mass units. The consistent correlations between the reflectance data and biochemical parameters indicate the success of the model in predicting and validating these parameters in the leaves of the study plants (Table 2).

**Figure 4:**
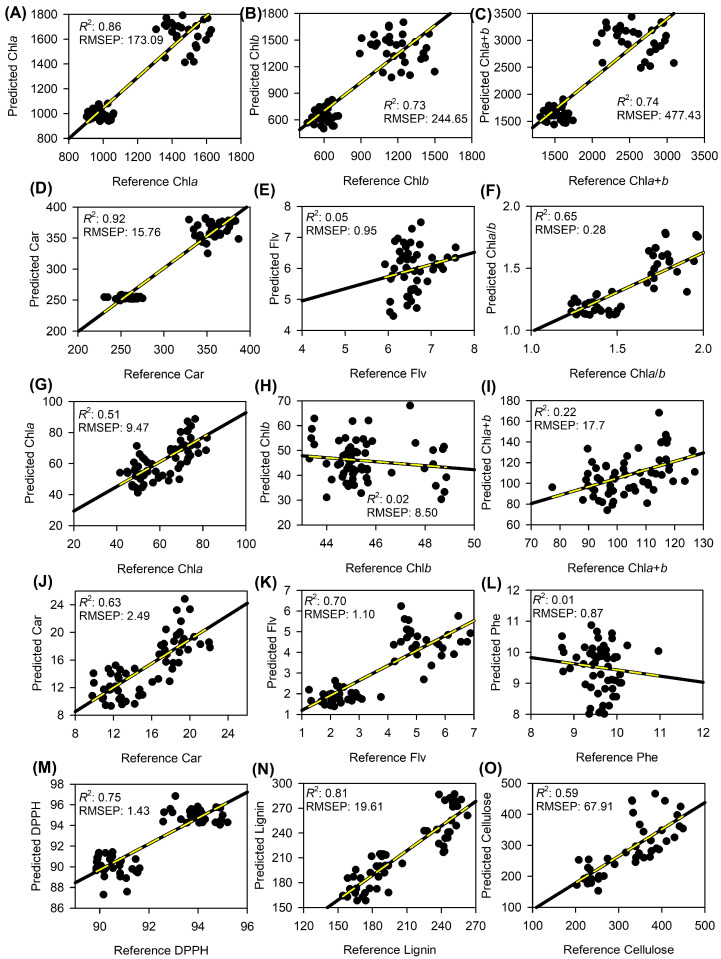
Scatter plots of reference versus predicted data using partial least squares regression (PLSR) for various biochemical parameters in leaves. The dotted yellow lines represent the regression fit, and the solid black lines denote the 1:1 line. Model performance metrics, including the coefficient of determination (R^2^) and root mean square error of prediction (RMSEP), were provided. The models were trained using 70% of the data for calibration and validated using the remaining 30%. Bias values were consistently below 0.01 and are not shown. (**A**) Chlorophyll a-based area (Chl*a*(area)), (**B**) chlorophyll *b*-based area (Chl*b*(area)), (**C**) combined chlorophyll *a*+*b*-based area (Chl*a*+*b*(area)), (**D**) carotenoid-based area (Car(area)), (**E**) flavonoid-based area (Flv(area)), (**F**) chlorophyll *a*/*b* ratio (Chl*a*/*b*), (**G**) chlorophyll *a*-based mass (Chl*a*(mass)), (**H**) chlorophyll *b*-based mass (Chl*b*(mass)), (**I**) combined chlorophyll *a*+*b*-based mass (Chl*a*+*b*(mass)), (**J**) carotenoid-based mass (Car(mass)), (**K**) flavonoid-based mass (Flv(mass)), (**L**) phenolic compounds (Phe), (**M**) radical scavenging activity (DPPH), (**N**) lignin, and (**O**) cellulose. (n = 60).

The validation and prediction phases further attested to the accuracy of the established models, with Partial Least Squares Regression (PLSR) offering key insights into the correlations between hyperspectral reflectance data and biochemical parameters in leaves (Table 3 and Figure 4). The root mean square error of prediction (RMSEP) values were notably insightful. The precision of these PLSR models was visually represented in scatter plots, as depicted in Figure 4. The correlation, slope, offset, and other predictive statistical parameters for various biochemical parameters in the leaves of both plants are summarised in Table 3. This table shows the maximum PLS factor, correlation coefficient (r), slope, offset, standard error of prediction (SEP), ratio of prediction to deviation (RPD), bias, and the linear equation relating the prediction to the calibration model (R^2^P).

Chlorophyll *a* (Chl*a* (mg m^−2^)) exhibited a remarkable correlation coefficient (r) of 0.93 and a robust RPD of 2.65. With a maximum PLS factor of 6, the model displayed a slope of 0.70, an offset of 303.9, and a SEP of 144.5, underpinning its efficiency in predicting Chl*a* concentrations with a bias of 97.2. On the other hand, chlorophyll *b* (Chl*b* (mg m^−2^)) and total chlorophyll (Chl*a*+*b* (mg m^−2^)) had correlation coefficients of 0.85 and 0.86, respectively. The PLSR models for these parameters offered insights into their concentrations with biases of 124.2 and 286. Carotenoids (Car (mg m^−2^)), an essential capacity for photoprotection in plants, stood out with an exemplary r value of 0.96 and an RPD of 3.53, suggesting the model’s precision in predicting carotenoid concentrations. The Car (mg m^−2^) model, with a PLS factor of 6, displayed a slope of 0.90 and a low bias of 1.8, reinforcing its predictive accuracy.

In contrast, the flavonoids (Flv (nmol cm^−2^)) showed a lower correlation coefficient of 0.05. Even with a maximum PLS factor of 2, the prediction showed a SEP of 0.7 and a near-neutral bias of 0.6. However, when analysed on a different scale, such as Flv (μmol g^−1^), the correlation improved significantly to 0.83, with an RPD of 1.81, in agreement with the calibration models (R^2^ = 0.62). Phenolic compounds (Phe (mL L^−1^)) registered an RMSEP value of 1.35 but had a correlation coefficient of −0.11, indicating potential discrepancies in the predictions. Antioxidant capacity, represented by DPPH, also exhibited a strong correlation coefficient of 0.86, with an RPD value of 1.98, highlighting the model’s reliability for predicting antioxidant levels in plant samples.

Lignin (mg g^−1^), crucial for plant structural integrity, showcased notable performance with a correlation coefficient of 0.89 and an RPD of 2.24. The ability of this model to predict lignin concentrations with a PLS factor of 7 underscores its significance, given lignin’s pivotal role in plant physiology and its influence on plant reflectance spectra. For the cellulose PLSR model, with a maximum PLS factor of 5, the correlation coefficient (r) was 0.91, indicating a strong relationship between the predicted and observed cellulose concentrations. The model exhibited an RPD value of 2.54, demonstrating its reliability in predicting cellulose concentrations.

In addition, the PLSR models have proven to be an indispensable tool offering unparalleled precision and reliability in predicting a myriad of biochemical attributes in the leaves of the examined plants. The consistent and robust performance across various parameters testifies to the potential of hyperspectral non-imaging coupled with PLSR in plant chemometric parameters, as shown in Table 3 and Figure 4.

### 2.5. Spectral Weighted Coefficients and Loadings from PLSR Analysis

**Figure 5:**
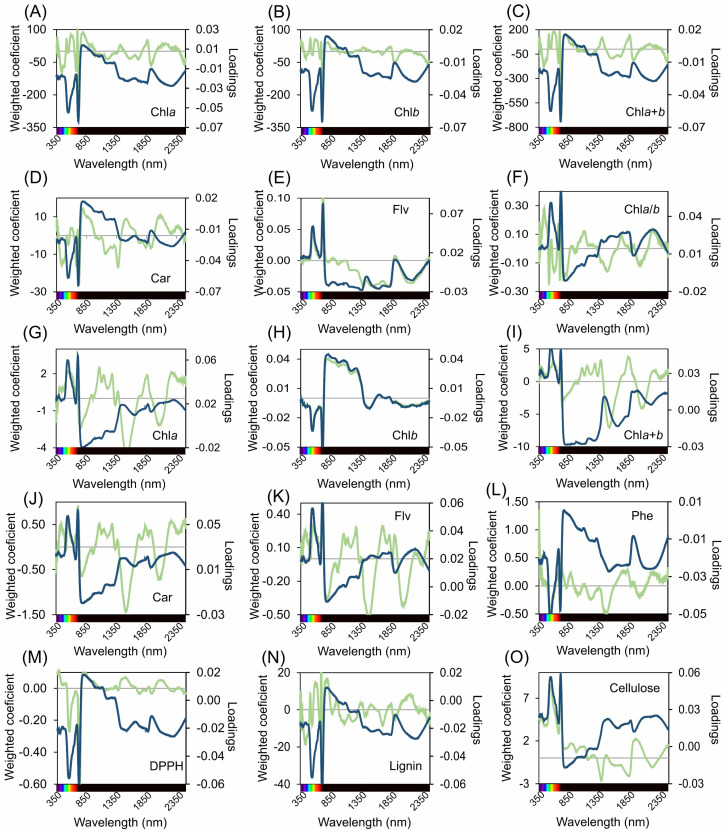
Weighted coefficients (green lines) and loadings (blue lines) from 350 to 2500 nm using partial least squares regression (PLSR) for various biochemical parameters in leaves. (**A**) Chlorophyll a-based area (Chl*a*(area)), (**B**) chlorophyll *b*-based area (Chl*b*(area)), (**C**) combined chlorophyll *a*+*b*-based area (Chl*a*+*b*(area)), (**D**) carotenoid-based area (Car(area)), (**E**) flavonoid-based area (Flv(area)), (**F**) chlorophyll *a*/*b* ratio (Chl*a*/*b*), (**G**) chlorophyll *a*-based mass (Chl*a*(mass)), (**H**) chlorophyll *b*-based mass (Chl*b*(mass)), (**I**) combined chlorophyll *a*+*b*-based mass (Chl*a*+*b*(mass)), (**J**) carotenoid-based mass (Car(mass)), (**K**) flavonoid-based mass (Flv(mass)), (**L**) phenolic compounds (Phe), (**M**) radical scavenging activity (DPPH), (**N**) lignin, and (**O**) cellulose.

In the PLSR model analysis, the metrics for the weight and loadings across the UV-VIS-NIR-SWIR spectral range are in Figure 5. The analysis indicated a consistent distribution of regions characterised by prominent peaks and valleys, underscoring the role of weights and loadings in the formulation of the accuracy and precision of the predictive model. A meticulous analysis of the PLSR model revealed one of the two salient wavelengths within the 350 to 2500 nm range, each intrinsically linked to specific biochemical molecules.

For Chl*a* (mg m^−2^), a pronounced peak wavelength was observed at 698 nm, complemented by a significant valley at 723 nm. In a similar leaf, Chl*b* (mg m^−2^) exhibited a peak at 515 nm and a valley at 723 nm. The wavelengths associated with Chl*a*+*b* (mg m^−2^) peak at 696 nm and a valley at 722 nm. Meanwhile, carotenoids (mg m^−2^) register a peak at 789 nm and a valley at 725 nm. The parameter flavonoids (nmol cm^−2^) is characterised by a peak at 719 nm and a valley at 1392 nm.

When transitioning to mass units, there is a discernible shift in wavelength. The Chl*a*/*b* (mg g^−1^) peaks at 721 nm and valleys at 516 nm. Chl*a* (mg g^−1^) peaks at 1064 nm with a valley at 1521 nm, whereas Chl*b* (mg g^−1^) peaks at 803 nm and valleys at 716 nm. The wavelengths for Chl*a*+*b* (mg g^−1^) peak at 698 nm and valley at 1521 nm, and those for carotenoids (mg g^−1^) peak at 716 nm with a valley at 1505 nm. The flavonoids (μmol g^−1^) also exhibited peaks at 715 nm and valleys at 1489 nm. Phenolics (mL L^−1^) were marked by a peak at 354 nm and a valley at 1468 nm, and DPPH peaks at 363 nm with a valley at 719 nm. The wavelengths for the mg de lignin g^−1^ peak at 698 nm and valley at 723 nm, and those for cellulose (nmol mg^−1^) peak at 713 nm and valley at 1383 nm (Figure 5).

The wavelengths identified in this study provide a profound understanding of the interplay between spectral data and molecular composition, paving the way for advanced insights into hyperspectroscopy and its contributions to specific modelling endeavours.

### 2.6. Hyperspectral Vegetation Index for Selected Most Responsive Wavelengths and Bands

**Figure 6:**
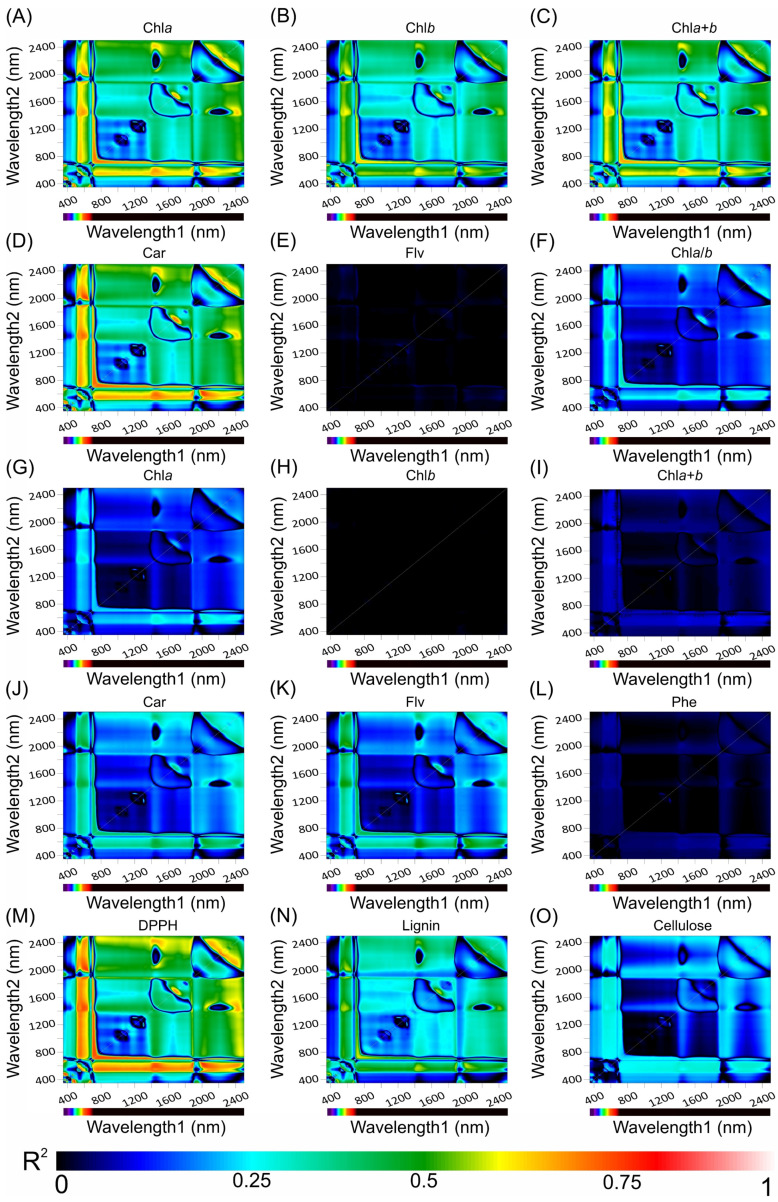
Counter map for correlation coefficients (R^2^) from HVI algorithms applied to reflectance hyperspectroscopy data across the range of 350 to 2500 nm. The heatmap demonstrates the coefficient of correlation (R^2^) obtained from linear regression analyses between various biochemical compounds and the interactions between Wavelengths1 and Wavelengths2. (**A**) Chlorophyll a-based area (Chl*a*(area)), (**B**) chlorophyll *b*-based area (Chl*b*(area)), (**C**) combined chlorophyll *a*+*b*-based area (Chl*a*+*b*(area)), (**D**) carotenoid-based area (Car(area)), (**E**) flavonoid-based area (Flv(area)), (**F**) chlorophyll *a*/*b* ratio (Chl*a*/*b*), (**G**) chlorophyll *a*-based mass (Chl*a*(mass)), (**H**) chlorophyll *b*-based mass (Chl*b*(mass)), (**I**) combined chlorophyll *a*+*b*-based mass (Chl*a*+*b*(mass)), (**J**) carotenoid-based mass (Car(mass)), (**K**) flavonoid-based mass (Flv(mass)), (**L**) phenolic compounds (Phe), (**M**) radical scavenging activity (DPPH), (**N**) lignin, and (**O**) cellulose. The colour gradient, transitioning from dark blue to light red, signifies increasing correlation strength.

Hyperspectral vegetation indices spanning the wavelength spectrum from 350 to 2500 nm and clear correlation dynamics were observed. The area based on chlorophyll *a* (Chl*a*(area)) stood out with a compelling (R^2^) value of 0.89, underscoring its pronounced linear association with the examined wavelengths. Conversely, several parameters, namely, the chlorophyll *a*/*b* ratio (Chl*a*/*b*), flavonoids (Flv), area, chlorophyll *a* mass (Chl*a*(mass)), combined chlorophyll *a*+*b* mass (Chl*a*+*b*(mass)), phenolic compounds (Phe), and cellulose, revealed milder correlations. The cluster heatmap gradient, transitioning seamlessly from deep blue to red, elegantly encapsulated these insights, presenting an intricate portrayal of correlation magnitudes across diverse biochemical constituents (Figure 6).

### 2.7. Algorithms for Selected Most Responsive Wavelengths and Bands

**Figure 7:**
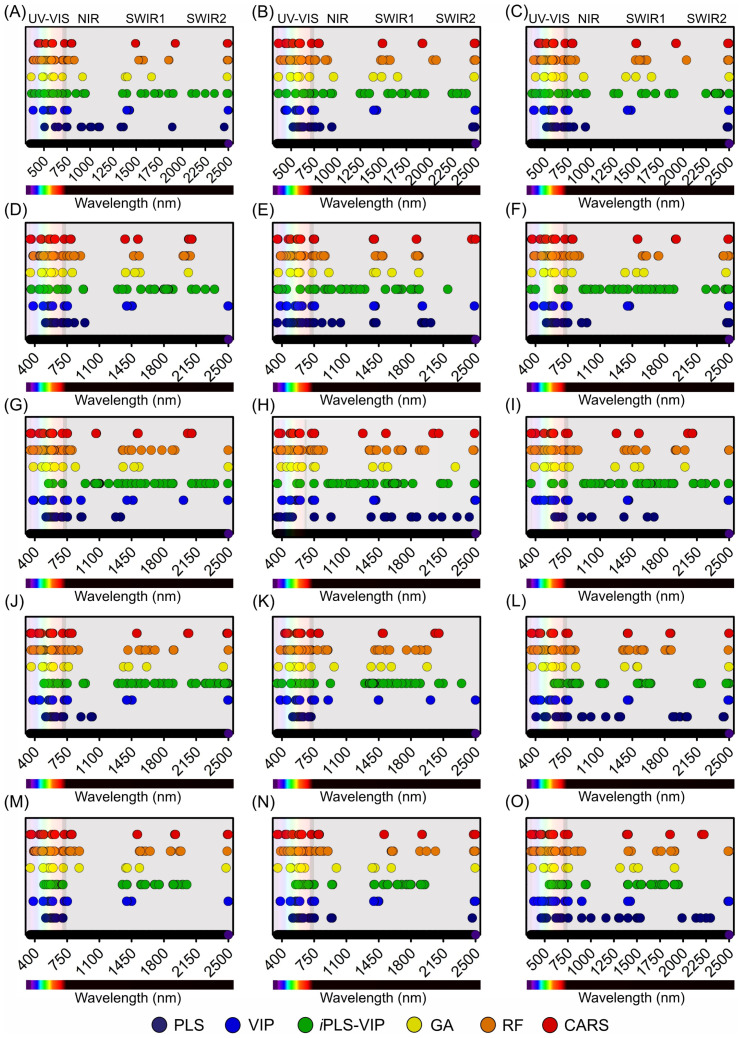
Selection of the most responsive variables across the wavelength range of 350–2500 nm (UV-VIS, NIR, SWIR1, SWIR2) using various algorithms, including partial least squares (PLS), variable importance in projection (VIP), interval PLS-VIP (*i*PLS-VIP), genetic algorithms (GA), random forest (RF), and competitive adaptive reweighted sampling (CARS), for Hibiscus and geranium plants. (**A**) Chlorophyll a-based area (Chl*a*(area)), (**B**) chlorophyll *b*-based area (Chl*b*(area)), (**C**) combined chlorophyll *a*+*b*-based area (Chl*a*+*b*(area)), (**D**) carotenoid-based area (Car(area)), (**E**) flavonoid-based area (Flv(area)), (**F**) chlorophyll *a*/*b* ratio (Chl*a*/*b*), (**G**) chlorophyll *a*-based mass (Chl*a*(mass)), (**H**) chlorophyll *b*-based mass (Chl*b*(mass)), (**I**) combined chlorophyll *a*+*b*-based mass (Chl*a*+*b*(mass)), (**J**) carotenoid-based mass (Car(mass)), (**K**) flavonoid-based mass (Flv(mass)), (**L**) phenolic compounds (Phe), (**M**) radical scavenging activity (DPPH), (**N**) lignin, and (**O**) cellulose.

**Figure 8:**
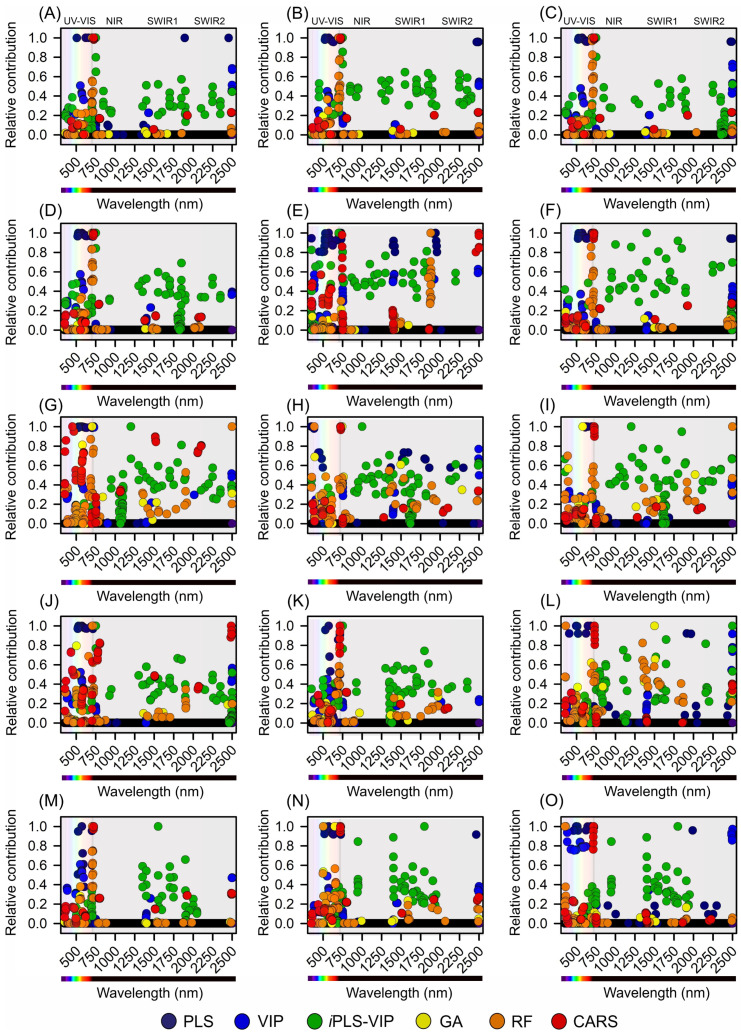
Relative contributions of the most responsive variables across the wavelength range of 350–2500 nm spanning the UV-VIS, NIR, SWIR1, and SWIR2 regions. Selection was performed using algorithms such as partial least squares (PLS), variable importance in projection (VIP), interval PLS-VIP (*i*PLS-VIP), genetic algorithms (GA), random forest (RF), and competitive adaptive reweighted sampling (CARS) for Hibiscus and geranium plants. (**A**) Chlorophyll a-based area (Chl*a*(area)), (**B**) chlorophyll *b*-based area (Chl*b*(area)), (**C**) combined chlorophyll *a*+*b*-based area (Chl*a*+*b*(area)), (**D**) carotenoid-based area (Car(area)), (**E**) flavonoid-based area (Flv(area)), (**F**) chlorophyll *a*/*b* ratio (Chl*a*/*b*), (**G**) chlorophyll *a*-based mass (Chl*a*(mass)), (**H**) chlorophyll *b*-based mass (Chl*b*(mass)), (**I**) combined chlorophyll *a*+*b*-based mass (Chl*a*+*b*(mass)), (**J**) carotenoid-based mass (Car(mass)), (**K**) flavonoid-based mass (Flv(mass)), (**L**) phenolic compounds (Phe), (**M**) radical scavenging activity (DPPH), (**N**) lignin, and (**O**) cellulose.

To determine the most responsive and relative contribution wavelengths spanning the UV-VIS-NIR-SWIR1-SWIR2 spectral regions for the Hibiscus and Geranium species, a comprehensive suite of advanced computational algorithms was employed. Partial least squares (PLS), variable importance in projection (VIP), interval PLS-VIP (*i*PLS-VIP), genetic algorithms (GA), random forest (RF), and competitive adaptive reweighted sampling (CARS), each with its distinct computational framework, provided a multifaceted perspective on spectral data (Figure 7 and Figure 8).

The partial least squares (PLS) technique demonstrated a notable affinity towards the UV and VIS regions. In the UV spectrum, a select cohort of 4 wavelengths emerged as pivotal, while in the VIS spectrum, a more expansive set of 131 wavelengths was demarcated. The NIR domain, rich in spectral information, is marked by 66 distinct wavelengths. Concurrently, the spectral behaviours of the SWIR1 and SWIR2 regions are often complex, with selections of 26 and 29 wavelengths, respectively. The variable importance in the projection (VIPs) algorithm, with its nuanced computational mechanics, unveiled a more expansive spectral selection. Within the UV domain, 57 wavelengths are accentuated. The VIS and NIR regions, both intricate in their spectral compositions, were densely populated, with wavelengths of 316 and 154, respectively. The SWIR1 and SWIR2 spectra were not overshadowed, with 133 and 75 wavelengths earmarked orderly. The integrated *i*PLS-VIP approach, which combines the principles of PLS and VIP, produces a diverse and intricate selection matrix. The UV and VIS domains were punctuated at wavelengths of 39 and 157, respectively. The NIR region, with its rich spectral intricacies, resonates profoundly with 197 selected wavelengths. The SWIR sectors, particularly SWIR1 and SWIR2, are delineated at wavelengths of 335 and 187, respectively.

Genetic algorithms (GAs), lauded for their dynamic computational adaptability, etched a distinct bias towards the UV and VIS domains, earmarking 15 and 50 wavelengths, respectively. The subsequent spectral niches, notably NIR, SWIR1, and SWIR2, were populated with 27, 44, and 14 wavelengths, respectively, thereby demonstrating the versatility of the algorithm.

The random forest (RF) algorithm, renowned for its robust equitability in data handling, unveiled a harmonious spectral distribution for model construction. It encompasses an array of 75 wavelengths in the UV domain, a robust contingent of 433 in the VIS spectrum, and a hearty 202 in the NIR bands. The SWIR spectra, with their unique spectral signatures, were carefully addressed, with SWIR1 and SWIR2 contributing 134 and 71 wavelengths, respectively.

In the last analysis, the Competitive Adaptive Reweighted Sampling (CARS) algorithm, with its intricate computational schema, presented a holistic spectral panorama. It identified 48 wavelengths in the UV bands, a substantial 320 in the VIS spectrum, 199 in the NIR bands, and a synergistic total of 243 spanning the SWIR1 (108) and SWIR2 (135) bands. All selected wavelengths were distributed for the evaluated biochemical parameters (Figure 7A–O).

## 3. Discussion

### 3.1. Biochemical Parameters

Understanding the biochemical parameters of plants provides insights into their physiological status, overall health, and responses to environmental stress. In this study, the parameters for *Hibiscus rosa-sinensis* L. (Hibiscus) and *Pelargonium zonale* (L.) L’Hér. Ex. Aiton (Geranium) was methodically assessed to predict and select the most responsive wavelengths and bands.

Chlorophylls, specifically chlorophyll *a* (Chl*a*) and chlorophyll *b* (Chl*b*), are the key pigments facilitating photosynthesis. For the plants studied, the average Chl*a* concentration (1322.8 mg m^−2^ and 64.2 mg g^−1^) was higher than that of Chl*b* (1012.0 mg m^−2^ and 46.0 mg g^−1^), in coherence with the known predominant presence of Chl*a* in plants. The higher coefficients of variation (CVs) associated with Chl*b* suggest greater variability, which can be attributed to the role of Chl*b* in adjusting the light absorbed for mechanisms of dissipation exceeding energy due to its broader absorption peak, as suggested by [19,20,21,22]. Along these lines, carotenoids (Cars) with higher accumulation play an integral role in photoprotection. These pigments are essential for safely dissipation of excess energy, particularly under intense light or stress conditions. Additionally, carotenoids assist in maintaining the structural integrity of the photosynthetic apparatus and act as antioxidants, protecting plant cells from potential damage caused by reactive oxygen species [4,23,24]. Their accumulation indicates a plant’s adaptive response to ensure optimal photosynthetic efficiency and minimise photodamage under varying environmental conditions.

Flavonoids are secondary metabolites recognised to protect plants against UV radiation and pathogens [20,25]. Their variable concentrations, as denoted by the high CV, possibly reflect the adaptive nature of plants to varying environmental factors. The consistently low CV for DPPH, an indicator of antioxidant potential, suggested that the radical scavenging capacity was relatively stable across the samples studied. This aligns with previous findings wherein plants exhibited consistent antioxidant capabilities despite varying conditions [5,26,27].

Lignin and cellulose are vital components of the plant cell wall, imparting structural integrity. The lower CV of lignin compared to cellulose suggests a more uniform distribution or a consistent synthesis mechanism across both species. Bloem, Gerighausen, Chen & Schnug (2020) [28] suggested that lignin biosynthesis is intricately regulated by mechanisms related to light interaction with the leaves, which could account for the observed consistency.

The correlative matrix and principal component analysis shed light on the intricate interactions between these biochemical parameters. The strong positive associations between lignin and chlorophyll parameters are consistent with those reported in previous studies. For instance, Vanholme, Demedts, Morreel, Ralph & Boerjan (2010) [29] proposed that lignin synthesis might be affected by the rate of photosynthesis and, consequently, chlorophyll content.

Moreover, the negative correlation between DPPH and the Chl*a*/*b* ratio and Flv might suggest a compensatory mechanism wherein higher antioxidant potential is associated with a lowered Chl*a*/*b* ratio, perhaps indicating stress conditions where Chl*a* predominance is essential. Carotenoids, which play a crucial role in photoprotection and are precursors for abscisic acid, show significant correlations with various parameters. Their positive association with Chl*a*, as noted by Steidle Neto et al. (2017) [21], is a testament to their synergistic role in photosynthesis.

Finally, the PCA results encapsulated 63.4% of the variance. They revealed distinctive biochemical compositions for Hibiscus and Geranium, reiterating species-specific biosynthetic pathways and regulatory mechanisms that differentiate plant species in terms of their biochemical constituents.

This exploration of the biochemical parameters of Hibiscus and Geranium leaves offers a comprehensive overview of their physiological and biochemical characteristics and underscores the intricate interplay of these parameters. These findings pave the way for further investigation into how alterations in plant biochemistry can modify the selected wavelengths and the most responsive bands.

### 3.2. Advanced Data Analysis for Hyperspectroscopy UV-VIS-NIR-SWIR

Hyperspectral reflectance is an efficient and effective method to discern spectra through imaging or non-imaging methods. It is a powerful tool that can capture and analyse information across various electromagnetic wavelengths. Its application in plant biochemistry, particularly in the UV-VIS-NIR-SWIR range, has grown significantly over the past decade because of its ability to provide detailed insights into the biochemical and structural properties of plant tissues without causing harm [1].

Spectral reflectance and its significance, for example, the spectral reflectance values for *Hibiscus rosa-sinensis* L. (Hibiscus) and *Pelargonium zonale* (L.) L’Hér. Ex. Aiton (Geranium), as captured across the UV-VIS-NIR-SWIR bands, revealed inherent biochemical differences between the two plant species. Transitions noted at 700 and 1300 nm are crucial, marking the shifts from the VIS spectrum to NIR and NIR to SWIR. These transitions, particularly from VIS to NIR, are often associated with the chlorophyll absorption peak, which provides insight into the photosynthetic efficiency of plants [30]. The marked difference in reflectance values, as corroborated by the t test, underscores the intrinsic biochemical variance between Hibiscus and Geranium. These differences may be attributed to variations in the chlorophyll content, cellular structures, and moisture content [31].

For example, PCA for hyperspectral analysis is an efficient way to analyse the data derived from curves. The utility of PCA in analysing hyperspectral data cannot be overstated for some of these aspects, such as CA and spectral diversity. As demonstrated by the significant variance captured by PC1 (83%) and PC2 (15%) and the high accuracy and precision, the technique effectively consolidates complex spectral data into digestible formats. Impressively, only two principal components accounted for nearly 98% of the variance, emphasising the distinct spectral characteristics of Hibiscus and Geranium plants. These spectral differences can be attributed to variations in compounds such as flavonoids, chlorophylls, and phenolic compounds, each with unique reflectance and absorption profiles in the hyperspectral range [32]. Moreover, the dominance of PC1 and PC2, as visually emphasised by the cumulative variability circles, further emphasises the robustness of PCA in representing the complex interplay of spectral wavelengths. Therefore, any slight variations in the third and subsequent principal components, which account for only a negligible portion of the total variance, are not expected to play a pivotal role in deciphering the overall biochemical and structural attributes of the two plants.

In this sense, complemented by advanced data analysis techniques such as PCA, hyperspectral sensors offer a promising avenue to decipher the complex biochemical attributes of photosynthetic pigments and other compounds in plants. As demonstrated by the distinct reflectance profiles of Hibiscus and Geranium, this technology holds significant potential for distinguishing plant species, understanding their unique biochemical compositions, and gaining insights into their physiological and structural properties.

### 3.3. Biochemical Parameters for Calibration, Cross-Validation, and Prediction PLSR Models

Partial least squares regression (PLSR) has proven to be a robust method for establishing relationships between hyperspectral reflectance data and various biochemical parameters in plants [18,33,34]. This study is no exception, where PLSR was employed to calibrate and cross-validate the relationships between reflectance data and biochemical parameters in the leaves of Hibiscus and *Pelargonium geranium* plants.

During the calibration phase, based on area, the chlorophyll *a* and carotenoid concentrations showed strong correlations with values of 0.93 and 0.96, respectively. Such high calibration values typically underscore a reliable model; however, as with most models, cross-validation often provides slightly lower correlation values. This study corroborates this expectation with values (R^2^) of 0.90 and 0.89 for chlorophylls and dissipation energy based on area and mass, respectively [35,36].

While chlorophyll *a* and *b* and carotenoids received significant attention in the study, less commonly studied biochemical parameters such as lignin concentrations also showed respectable calibration values. The calibration values for lignin, a complex organic polymer critical for structural support in vascular plants [37], were 0.74 and 0.71 for R^2^ to cross-validation, indicating reasonable model reliability.

The data for parameters such as DPPH, which represents the radical scavenging potential, along with cellulose and SAE, indicate the study’s efforts to achieve a comprehensive understanding of the biochemistry of plants. Nevertheless, the apparent need for further research or data acquisition regarding these parameters highlights the challenges faced when attempting to calibrate and validate models for certain biochemical compounds. This reflects a larger issue in the scientific community, where achieving high calibration values for certain parameters remains elusive even with advanced techniques [21,38,39].

Table 2 shows the calibration and cross-validation statistics for various biochemical parameters. It is essential to note that the ratio of prediction to deviation (RPD) values is invaluable because they provide insight into the quality of the calibration models. Typically, an RPD value greater than 2 indicates that a model is suitable for predictive purposes [40].

The validation and prediction phases further demonstrated the efficacy of the PLSR models. The high correlation coefficient (r) values, especially for photosynthetic pigments (0.85 to 0.96), signify a strong relationship between the observed and predicted values. The strong performance of carotenoids, essential compounds for photoprotection in plants [41], was particularly noteworthy, with an r value of 0.96.

However, not all the parameters showed stellar results. Flavonoids, for instance, demonstrate lower correlation values, underscoring the potential challenges in predicting certain biochemical parameters using hyperspectral data [42,43]. In contrast, lignin exhibited a reasonable r-value of 0.89.

The presented data underscore the potential and challenges of employing PLSR models to predict plant biochemical parameters using hyperspectral sensors. The efficacy of this technique in predicting a plethora of parameters, from chlorophyll to lignin, highlights its importance in modern plant research and potential applications in precision agriculture, phenotyping, and other related fields.

### 3.4. Selected Most Responsive Wavelengths and Bands for Algorithms and Molecular Insights

Exploration of the UV-VIS-NIR-SWIR spectral range yielded notable peaks and valleys, which serve as critical indicators of the correlation between specific wavelengths and unique biochemical molecules in plants. Such correlations underscore the importance of these wavelengths in determining the concentrations of the corresponding biochemical molecules in plant tissues. This is evident when observing the sensitivity of the red-edge region (approximately 690–730 nm) to chlorophyll forms Chl*a*, Chl*b*, and Chl*a*+*b*. These observations are well supported by the literature, including those of Gitelson and Solovchenko (2018) [44]. Similarly, the sensitivity of specific wavelengths to pigments such as carotenoids and flavonoids aligns with the results of Blackburn (2007) [45].

The hyperspectral vegetation index further emphasises the correlation between spectral data and vegetation properties. A notable observation here is the strong linear association between the photosynthetic concentration, structural molecules, and antioxidant compounds and the studied wavelengths, showing the efficacy of hyperspectral sensors as a non-invasive method, in line with the findings of Chen et al. (2019) [46].

Diving deeper into wavelength selection, it was observed that different computational algorithms offered varied perspectives on the most responsive wavelengths for both Hibiscus and Geranium species. For example, the partial least squares (PLS) method demonstrated a strong affinity for the UV and VIS regions, reinforcing its significance in determining biochemical concentrations. These data were consistent with those of Thenkabail et al. (2011) [33]. The variable importance in the projection (VIP) method showcased an expansive spectral selection, highlighting especially the VIS and NIR regions, which have been previously recognised for their role in determining water content and cellular structures by Kycko, Zagajewski, Lavender & Dabija (2019) [47]. Other methods, such as genetic algorithms (GAs) and random forest (RF), provide unique interpretations of the spectral data, with GAs leaning more towards the UV and VIS regions and RF offering a broader perspective. Finally, with its intricate mechanics, Competitive Adaptive Reweighted Sampling (CARS) captures a comprehensive spectral view, highlighting the value of a holistic spectral approach for deciphering vegetation biochemistry.

Understanding these correlations and the resulting insights from hyperspectroscopy will enhance our knowledge of plant hyperspectroscopy. This foundational understanding is crucial for developing advanced models that can predict biochemical content from hyperspectral data. The robust correlations between spectral data and molecular compositions demonstrate the high potential of hyperspectroscopy in precision agriculture, ecology, and environmental monitoring [36,48,49]. Given its ability for quick, non-invasive, and detailed evaluations, hyperspectroscopy has emerged as a pivotal tool and a promising timely intervention to ensure optimal plant health and productivity.

The interplay between spectral analysis and advanced computational algorithms has opened new avenues in hyperspectroscopy, highlighting its potential for mapping plant biochemical parameters effectively.

## 4. Materials and Methods

### 4.1. Experimental Design and Growth Conditions of Plants

**Figure 9:**
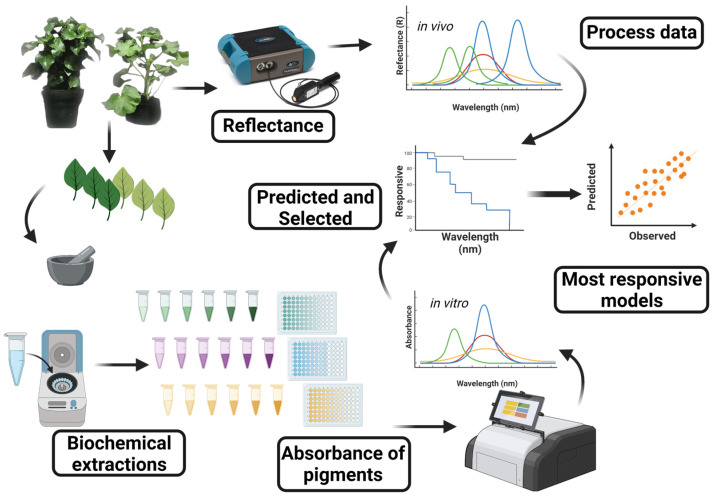
Flowchart of the methodology for assessing biochemical molecules in Hibiscus and Geranium leaves using UV-VIS-NIR-SIR hyperspectral sensors. Plants were cultivated in a greenhouse, and hyperspectral reflectance measurements of the leaves were taken. Biochemical extraction of pigments and cellular components was subsequently analysed using ELISA. Data from hyperspectral reflectance and biochemical absorbance were integrated and examined using PLS regression models. Responsive wavelengths were selected, and the corresponding PLS models were generated.

*Hibiscus rosa-sinensis* L. (Hibiscus) and *Pelargonium zonale* (L.) L’Hér. Ex. Aiton (Geranium) plants were cultivated in the Botanical Garden at the State University of Maringá, Maringá, Paraná, Brazil, under greenhouse conditions. These conditions provided natural ambient light, with temperatures between 22 °C and 26 °C, and a photoperiod of 16 h. To ensure consistent hydration, the plants were watered twice daily, at 8 a.m. and 6 p.m. Leaves of various ages were sampled from different parts of the plant. A total of 200 samples were collected for hyperspectral reflectance analysis and assessment of leaf biochemical profiles. To guarantee uniformity in the data collection, all measurements were conducted between 11 a.m. and 1 p.m. A schematic of the flowchart analysis is shown in Figure 9.

### 4.2. Acquisition of Hyperspectral Leaf Reflectance

Leaf hyperspectral reflectance was acquired using a FieldSpec^®^ 3 spectroradiometer complemented by an ASD contact PlantProbe^®^ (Analytical Spectral Devices ASD Inc., Boulder, CO, USA). The spectroradiometer incorporated three sensors spanning wavelengths ranging from 350 to 2500 nm. By employing the PlantProbe^®^, we ensured that the data remained uncontaminated by atmospheric interference. The measurements were directed at the adaxial surface of the leaves, deliberately avoiding the central vein. Periodic calibration of the device was conducted using a standard white reference plate (Spectralon^®^, Labsphere Inc., Longmont, CO, USA), resulting in 2151 bands within the 350–2500 nm spectrum. This method produced 200 distinctive hyperspectral leaf profiles aligned with the respective biochemical metrics. Optimal bands for chemometric evaluations were identified through principal component analysis and specific algorithms that discerned the most responsive wavelengths.

### 4.3. Profiling of Biochemical Compounds

To quantify the levels of total chlorophyll (Chl), carotenoids (Car), anthocyanins (AnC), and flavonoids (Flv) in the leaf extracts, we adopted a modified methodology based on Gitelson and Solovchenko (2018) [44]. Leaf samples, each 1 cm^2^, were homogenised in 2 mL tubes using a chloroform and methanol mixture (2:1 *v*/*v*) supplemented with CaCO_3_. After thorough extraction, we added distilled water, equivalent to 20% of the volume of the extract, to facilitate the separation of the polar and nonpolar phases. This solution was centrifuged at 15,000 rpm for 9 min to ensure a distinct phase division. For quantification, we placed a 200 µL aliquot of the extract into a quartz glass UV 96-well microplate. The resultant readings were acquired using the Biochrom Asys UVM-340 Microplate-Reader, complemented by the ScanPlus VisibleWell^®^ software version 1.0.2 (Biochrome Ltd., Milton Road, Cambridge, UK). Furthermore, leaf segments utilised for extraction quantification were oven-dried at 70 °C until they reached a constant weight. Subsequent measurements were performed using an analytical balance to express the results per unit of mass.

#### 4.3.1. Chlorophyll and Carotenoid Quantification

To quantify chlorophyll *a*, *b*, *a*+*b*, and carotenoids (carotenes and xanthophylls), 200 µL of methanolic extract was added to each well. Absorbance was recorded at 470, 652, and 665 nm using a methanol extract. The formulae presented by Falcioni et al. (2023) [35] were used to determine the chlorophyll and carotenoid concentrations expressed in mg cm^−2^ and mg g^−1^.

#### 4.3.2. Flavonoid and Anthocyanin Quantification

The polar fraction of the methanolic extract was analysed to assess flavonoid (Flv) concentrations. The absorbance of these extrachloroplastidic pigments was determined at λ358 nm using a molar absorption coefficient of ε358 = 25 mM^−1^ cm^−1^, as described by Gitelson & Solovchenko (2018) [44]. After Flv quantification, the water-methanol phase was acidified with hydrochloric acid to a final concentration of 0.1% HCl. This adjustment facilitated the determination of anthocyanin (AnC) levels at λ530 nm, employing a molar absorption coefficient of ε530 = 30 mM^−1^ cm^−1^, as reported by Gitelson et al. (2020) [41].

#### 4.3.3. Total Soluble Phenolic Compounds

Soluble phenolic compounds (PhCs) were quantified using a modified procedure of Ragaee (2006) [50]. For this assay, a 2 mL Eppendorf tube was loaded with 150 μL of the methanolic extract, 70 μL of 1 M Folin–Ciocalteu reagent, 140 μL of 3.56 M Na_2_CO_3_, and 850 μL of deionised water. Following a 50-min incubation in the dark, the mixture was centrifuged at 15,000 rpm for 2 min. The absorbance of the supernatant was measured at λ725 nm using a quartz glass microplate reader. Gallic acid served as the standard for estimating the equivalent Phe concentration, characterised by the equation Ŷ = 87.651x + 1.6515 with an R^2^ value of 0.993.

#### 4.3.4. Antioxidant Compounds

The antioxidant potential was determined using the DPPH (2,2-diphenyl-1-picrylhydrazyl) free radical neutralisation method, adapted from the protocol outlined by Llorach et al. (2008) [27]. DPPH solution (1 mM) was used in this assay. The reaction was initiated by adding 50 µL of the methanolic extract to 200 µL of DPPH solution. After vigorous mixing, the samples were incubated in darkness for an hour. Absorbance measurements were performed using a quartz glass 96-well microplate reader at λ515 nm [34].

### 4.4. PLSR Analysis of UV-VIS-NIR-SWIR Reflectance in Plants

For PLSR analysis, the dataset was divided into two subsets: 140 samples for calibration and cross-validation, and an additional 60 samples were designated for the external validation of the model. Multiple plant biochemical parameters were assessed, including the area- and mass-based metrics of chlorophyll *a*, chlorophyll *b*, total chlorophyll *a*+*b*, carotenoids, flavonoids, chlorophyll *a*/*b* ratio, phenolic compounds, lignin, and cellulose. These parameters were compared with the UV-VIS-NIR-SWIR spectral curves, considering each to be an independent entity. PLSR models were developed using the NIPALS algorithm. Outliers were identified using Leverage’s type and further examined using Leverage and Hotelling’s T^2^ methods with a threshold set at 5%. The performance of the model was evaluated using the coefficients of determination (R^2^) and the root mean square error (RMSE) across the calibration, cross-validation, and prediction stages. Based on benchmarks established by Minasny et al. (2013) [51], R^2^ values above 0.75 indicated optimal model performance, those between 0.75 and 0.5 were considered adequate, and values below 0.5 indicated suboptimal predictions. Additionally, the ratio of performance to deviation (RPD) was derived from the R^2^ values across different stages, providing insights into the precision of PLS model predictions. Calibration, cross-validation, and validation statistics for two plant species: PLS factors, R^2^ values, offset, RMSE, and RPD during the calibration and cross-validation stages for each parameter, as well as predictive statistics such as the correlation coefficient (R^2^), slope, offset, SEP, RPD, and the equation linking prediction to the calibration model [52].

### 4.5. Evaluating Hyperspectral Vegetation Indices Using Optimal Wavelengths

To optimise the accuracy of biochemical assessments, key hyperspectral bands were identified using the normalised difference vegetation index formula (Equation (1)) drawn from Crusiol et al., (2023) [53]. This approach generated distinct hyperspectral vegetation indices (HVIs). Each HVI was correlated with cross-sections relevant to phenomenological flows. Correlations were quantified using the Pearson correlation coefficient and coefficient of determination (R^2^) using the custom IDL code. A ground-based sensor captured spectra from 350 to 2500 nm, and the results are depicted in a contour map.

(1) $$HVI=\frac{Wavelength\;1-Wavelength\;2}{Wavelength\;1+Wavelength\;2}$$

### 4.6. Algorithmic Determination of Key Wavelengths in Plants

To accurately discern the most relevant wavelengths for our investigations of the Hibiscus and Geranium plants, a suite of advanced algorithms was utilised. It incorporates techniques such as partial least squares (PLS), variable importance in projection (VIP), interval PLS-VIP (*i*PLS-VIP), genetic algorithms (GA), random forests (RF), and competitive adaptive reweighted sampling (CARS). Data analysis was performed with precision using multiple software platforms. The R software package version 4.2.2 Corrplot R-Core Team 2021 and the Python programming language version 3.11.5 (Python Software Foundation, Wilmington, DE, USA) formed the foundation of our analytical framework. In Python, RF procedures were facilitated by the scikit-learn library, whereas the DEAP library underpinned our GA evaluations. In the R environment, the PLS package was paramount for the PLS-focused analyses. Additionally, for *i*PLS analyses, MATLAB 2022a software version 9.12 (MathWorks, Inc., Natick, MA, USA) was used and seamlessly integrated with PLS_Toolbox (Eigenvector Research, Inc., Manson, WA, USA). The relative contribution of each wavelength was determined by identifying the most responsive wavelengths. This was based on the maximum and minimum values selected by the wavelength selection algorithms.

### 4.7. Statistical Analyses

#### 4.7.1. Descriptive, Univariate and Multivariate Statistical Analyses

Comprehensive descriptive statistics were used to characterise the biochemical metrics. For each parameter, evaluations included count (n), mean, median, minimum, maximum, and coefficient of variation (CV, %), as delineated by [4]. The categorisation of CV adhered to the criteria proposed by Zar (2010) [54]. Pearson’s correlation coefficient was used to determine the interrelationships between biochemical attributes. For these analytical tasks, we used Statistica 10^®^ (StatSoft Inc., Tulsa, OK, USA) and the R software framework. Graphical depictions were generated using a suite of applications: SigmaPlot 10.0^®^ (Systat Inc., Santa Clara, Silicon Valley, CA, USA), specific R packages, Excel (Microsoft Inc., Silicon Valley, CA, USA), and CorelDraw 2020^®^ (Corel Corp., Ottawa, ON, Canada).

#### 4.7.2. Principal Component Analysis (PCA)

The Unscrambler X software, version 10.4 (CAMO Software, Oslo, Norway), was used to conduct PCA on the growth parameter data, with a statistical significance level set at *p* < 0.01. To avoid underfitting and overfitting, the optimal number of principal components was determined based on the first maximum value of overall accuracy [25].

## 5. Conclusions

These findings demonstrated the UV-VIS-NIR-SWIR spectral range, revealing its cardinal role in identifying the distinctive biochemical constituents of Hibiscus and Geranium plants. The reliability of our models was exemplified by R^2^ values consistently surpassing the 0.75 threshold, reinforcing the red edge in predicting vital plant molecules, such as chlorophyll. Additionally, parameters such as DPPH and lignin yielded significant outcomes, achieving R^2^ values of 0.86 for DPPH and 0.89 for lignin. Our application of advanced algorithms, particularly PLS, VIP, CARS, and other models, indicates an intricate relationship between the spectral data and plant biochemistry. The identification of highly responsive wavelengths, particularly in the red-edge region, emphasises deep-seated correlations with key plant pigments. Finally, the fusion of hyperspectroscopy and cutting-edge computational methodologies holds great promise in the future. This signifies a new era in precision agriculture and environmental oversight. Furthermore, they reduce the costs of reagents and their environmental disposal, thereby contributing to sustainability. Finally, chemometric methods applied to hyperspectral analysis are good predictive tools. The extensive yet largely untapped potential of hyperspectroscopy, as presented in our study, can be used for further exploration, fostering an environment ripe for innovation and transformative advances in sustainable agricultural practices.

## Figures and Tables

**Table 1:** Descriptive statistics of biochemical parameters measured in leaves of *Hibiscus rosa-sinensis* L. (Hibiscus) and *Pelargonium zonale* (L.) L’Hér. Ex. Aiton (Geranium). For each parameter, the table presents the count (n), mean, median, minimum, maximum, and coefficient of variation (CV %). (n = 200).

Parameters	Count (n)	Mean	Median	Minimum	Maximum	CV (%)
Chl*a* (mg m^−2^)	200	1322.8	1162.8	655.3	2089.0	29.0
Chl*b* (mg m^−2^)	200	1012.0	977.3	233.5	2222.1	47.0
Chl*a*+*b* (mg m^−2^)	200	2334.8	2141.3	999.9	4311.1	36.7
Car (mg m^−2^)	200	309.6	263.8	225.8	423.1	19.9
Flv (nmol cm^−2^)	200	6.5	6.3	2.3	25.0	31.8
Chl*a*/*b* ratio	200	1.5	1.3	0.9	3.7	29.5
Chl*a* (mg g^−1^)	200	64.2	61.2	28.4	134.2	27.0
Chl*b* (mg g^−1^)	200	46.0	44.6	14.9	114.7	31.4
Chl*a*+*b* (mg g^−1^)	200	110.2	106.6	43.3	249.0	26.2
Car (mg g^−1^)	200	15.6	14.8	6.3	32.9	32.4
Flv (μmol g^−1^)	200	3.6	3.0	0.7	12.2	60.0
Phe (mL L^−1^)	200	9.5	9.7	5.9	11.3	10.3
DPPH	200	92.6	93.3	86.2	99.6	2.9
Lignin (mg g^−1^)	200	216.7	213.5	125.4	297.3	19.7
Cellulose (nmol mg^−1^)	200	312.7	289.3	139.2	597.8	37.4

**Table 2:** Calibration and cross-validation validation statistics for biochemical parameters measured in leaves of *Hibiscus rosa-sinensis* L. (Hibiscus) and *Pelargonium zonale* (L.) L’Hér. Ex. Aiton (Geranium) using PLS regression models. The table presents the maximum PLS factor, coefficients of determination (R^2^), offset, root mean square error (RMSE), and ratio of prediction to deviation (RPD) for each parameter during the calibration and cross-validation. (n = 140).

Parameters	Maximum Factor PLS	Calibration				Cross-Validation			
		R^2^	Offset	RMSE	RPD	R^2^	Offset	RMSE	RPD
Chl*a* (mg m^−2^)	6	0.73	355.6	207.4	1.92	0.71	370.3	216.2	1.85
Chl*b* (mg m^−2^)	5	0.63	374.1	306.7	1.64	0.60	388.1	318.5	1.58
Chl*a*+*b* (mg m^−2^)	6	0.73	626.6	470.1	1.93	0.71	656.4	490.8	1.85
Car (mg m^−2^)	6	0.86	44.1	24.0	2.66	0.85	45.0	25.1	2.54
Flv (nmol cm^−2^)	2	0.08	6.0	1.7	1.04	0.04	6.2	1.7	1.02
Chl*a*/*b* ratio	7	0.49	0.7	0.3	1.41	0.42	0.8	0.3	1.31
Chl*a* (mg g^−1^)	5	0.33	43.1	13.9	1.22	0.27	44.9	14.6	1.17
Chl*b* (mg g^−1^)	1	0.12	39.2	12.6	1.07	0.01	41.8	13.4	1.00
Chl*a*+*b* (mg g^−1^)	4	0.14	92.4	24.8	1.08	0.08	96.4	25.9	1.04
Car (mg g^−1^)	5	0.49	7.9	3.4	1.39	0.43	8.3	3.6	1.32
Flv (μmol g^−1^)	5	0.62	1.3	1.2	1.62	0.57	1.4	1.3	1.53
Phe (mL L^−1^)	7	0.24	7.3	0.7	1.15	0.16	7.6	0.8	1.09
DPPH	4	0.81	17.4	1.1	2.30	0.80	17.8	1.2	2.22
Lignin (mg g^−1^)	5	0.74	57.7	21.1	1.96	0.71	60.9	22.3	1.85
Cellulose (nmol mg^−1^)	3	0.43	177.7	93.1	1.33	0.39	183.7	96.3	1.28

**Table 3:** Predictive statistical parameters obtained from PLS regression models for biochemical parameters in leaves of *Hibiscus rosa-sinensis* L. (Hibiscus) and *Pelargonium zonale* (L.) L’Hér. Ex. Aiton (Geranium). The table presents the maximum PLS factor, correlation coefficient (r), slope, offset, standard error of prediction (SEP), ratio of prediction to deviation (RPD), bias, and the linear equation relating prediction to the calibration model (R^2^P). (n = 140).

Parameters	Maximum Factor PLS	Predicted						
		r	Slope	Offset	SEP	RPD	Bias	Linear Equation Prediction to Calibration Model (R^2^_P_)
Chl*a* (mg m^−2^)	6	0.93	0.70	303.9	144.5	2.65	97.2	Ŷ = 1.2342x − 188.323
Chl*b* (mg m^−2^)	5	0.85	0.68	204.3	212.5	1.90	124.2	Ŷ = 1.0704x + 60.939
Chl*a*+*b* (mg m^−2^)	6	0.86	0.66	506.1	385.5	1.97	286.0	Ŷ = 1.1211x + 36.696
Car (mg m^−2^)	6	0.96	0.90	29.9	15.8	3.53	1.8	Ŷ = 1.0254x − 6.0335
Flv (nmol cm^−2^)	2	0.21	0.12	5.9	0.7	1.02	0.6	Ŷ = 0.3906x + 3.3941
Chl*a*/*b* ratio	7	0.80	1.00	0.2	0.2	1.67	0.2	Ŷ = 0.6383x + 0.3496
Chl*a* (mg g^−1^)	5	0.70	0.61	23.4	9.5	1.39	0.7	Ŷ = 0.7922x + 13.582
Chl*b* (mg g^−1^)	1	−0.14	−0.02	46.8	8.6	1.01	0.3	Ŷ = −0.7951x + 82.055
Chl*a*+*b* (mg g^−1^)	4	0.47	0.27	75.0	17.4	1.13	4.3	Ŷ = 0.8172x + 23.282
Car (mg g^−1^)	5	0.79	0.71	4.9	2.5	1.61	0.4	Ŷ = 0.8738x + 1.5158
Flv (μmol g^−1^)	5	0.83	0.96	0.7	0.9	1.81	0.6	Ŷ = 0.7221x + 0.4777
Phe (mL L^−1^)	7	−0.11	−0.06	10.1	0.9	1.01	0.1	Ŷ = −0.1992x + 11.423
DPPH	4	0.86	0.59	37.2	1.4	1.98	0.3	Ŷ = 1.2514x − 22.908
Lignin (mg g^−1^)	7	0.89	0.81	33.3	17.7	2.24	8.8	Ŷ = 0.994x + 10.078
Cellulose (nmol mg^−1^)	3	0.77	0.67	128.9	57.4	1.56	37.3	Ŷ = 0.8635x + 6.1253

## Data Availability

Not applicable.
